# Visual motion integration is mediated by directional ambiguities in local motion signals

**DOI:** 10.3389/fncom.2013.00167

**Published:** 2013-11-18

**Authors:** Francesca Rocchi, Tim Ledgeway, Ben S. Webb

**Affiliations:** School of Psychology, University of NottinghamNottingham, UK

**Keywords:** vision, global motion, local motion, spatial pooling, vector average, maximum likelihood, winner-take-all

## Abstract

The output of primary visual cortex (V1) is a piecemeal representation of the visual scene and the response of any one cell cannot unambiguously guide sensorimotor behavior. It remains unsolved how subsequent stages of cortical processing combine (“pool”) these early visual signals into a coherent representation. We (Webb et al., 2007, 2011) have shown that responses of human observers on a pooling task employing broadband, random dot motion can be accurately predicted by decoding the maximum likelihood direction from a population of motion-sensitive neurons. Whereas Amano et al. (2009) found that the vector average velocity of arrays of narrowband, two-dimensional (2-d) plaids predicts perceived global motion. To reconcile these different results, we designed two experiments in which we used 2-d noise textures moving behind spatially distributed apertures and measured the point of subjective equality between pairs of global noise textures. Textures in the *standard* stimulus moved rigidly in the same direction, whereas their directions in the *comparison* stimulus were sampled from a set of probability distributions. Human observers judged which noise texture had a more clockwise (CW) global direction. In agreement with Amano and colleagues, observers' perceived global motion coincided with the vector average stimulus direction. To test if directional ambiguities in local motion signals governed perceived global direction, we manipulated the fidelity of the texture motion within each aperture. A proportion of the apertures contained texture that underwent rigid translation and the remainder contained dynamic (temporally uncorrelated) noise to create locally ambiguous motion. Perceived global motion matched the vector average when the majority of apertures contained rigid motion, but with increasing levels of dynamic noise shifted toward the maximum likelihood direction. A class of population decoders utilizing power-law non-linearities can accommodate this flexible pooling.

## Introduction

One of the fundamental computational challenges faced by the visual system is to extract information about movement in the world from the patterns of light that form the retinal image. It is well-established that in the primate, motion sensitivity first emerges in primary visual cortex (area V1) where many visual neurons exhibit direction-selectivity to local edges and contours that move within their receptive fields. However, these neurons have relatively small receptive fields (e.g., Hubel and Wiesel, 1968) and consequently suffer from the “aperture problem,” in that the response of any one cell cannot convey the global (overall) direction of a spatially extensive object translating across the visual field. That is, neural responses based upon a purely local analysis of motion are inherently ambiguous and are potentially consistent with many possible real-world situations (Stumpf, 1911; Wallach, 1935). In principle, this ambiguity can be overcome by pooling the outputs of local motion detectors across space and over time. Although the site of this spatiotemporal pooling is consistent with the known properties of extrastriate cortical areas such as V5/MT, where motion-sensitive cells have receptive fields much larger (~100 times) than those in V1 (Gattass and Gross, 1981; Albright and Desimone, 1987), the precise nature of the global motion computation is still the subject of much uncertainty.

A variety of solutions have been proposed to account for the pooling of local motion signals in the human visual system. Early psychophysical studies tended to employ moving plaid patterns (composed of two, superimposed drifting gratings of different orientations) to investigate the computation underlying global motion perception. For example, Adelson and Movshon (1982) suggested that the visual system might integrate spatially one-dimensional (1-d) component motion signals into a global percept by utilizing the *intersection of constraints* (IOC) rule across different orientations. In this rule each 1-d component is represented as a vector orthogonal to its orientation and velocity constraint lines (indicating the range of possible velocities for each component), drawn perpendicular to each vector. These constraint lines intersect at a single point in velocity space that reveals the plaid's true motion. The IOC is a mathematically elegant solution to the problem of determining the global direction of a rigidly moving object from its spatially 1-d features, and computational models based on this principle have been developed (Simoncelli and Heeger, 1998). However, its complexity and inability to generalize to non-rigid movement (i.e., when the retinal image of an object deforms as it moves) cast doubt on its utility as a general model of human global motion processing. An alternative to the IOC, is the *vector average* (VA) or *vector sum*^1^ rule (Ferrera and Wilson, 1990; Wilson et al., 1992; Kim and Wilson, 1993), in which each spatially 1-d motion component is again represented as a vector orthogonal to its orientation and the two vectors are combined additively to determine the resultant direction of image motion. For example Yo and Wilson (1992) found that when two drifting gratings with identical spatial frequencies and contrasts but different speeds are superimposed to form a type II plaid pattern (which has different IOC and VA predictions), its perceived global direction at brief stimulus exposures (<60 ms), was readily predicted by the VA of the local directions. Similarly Wilson and colleagues have shown that the perceived direction of plaids constructed from two, second-order, contrast-modulated gratings is consistent with a VA computation of the directions of the individual components. Thus, for relatively simple narrowband stimuli such as plaids, the IOC and VA have been proposed as candidate solutions for the problem of encoding the global direction of the image. However, they are predominantly stimulus-based theories of global motion processing, albeit with different assumptions, based on rules rather than the known computations of the underlying neural mechanisms.

More recent psychophysical investigations of the computational principles of global motion processing in human vision have tended to employ broadband random-dot-kinematograms (RDKs) as stimuli. In RDKs a dense spatial array of randomly-positioned dots are displaced over space and time, according to a set of rules, to create the impression of net motion in a given direction. Williams and Sekuler (1984), for example, employed RDKs in which the individual dot directions were selected from a uniform probability distribution, such that each dot was assigned an independent, random walk in direction over time. They reported that provided that the range of local dot directions was constrained, the stimulus appeared to drift *en masse* in a global direction close to the VA of the dot directions. Several other studies (e.g., Watamaniuk and Duchon, 1992; Zohary et al., 1996) have also identified different stimulus-based summary statistics (e.g., mean or mode of the dot motions) which best characterize the perceived global direction or speed of RDKs. We (Webb et al., 2007) have recently shown, however, that when local dot directions are drawn from asymmetric probability distributions, image summary statistics are consistently poor predictors of perceived global motion direction. Instead mechanism-based, read-out algorithms, such as maximum-likelihood (ML) or winner-take-all (WTA) decoders, operating on the outputs of a simulated population of biologically-plausible, direction-selective neurons, offer a much more accurate and robust guide to human global motion perception (Webb et al., 2011). Given that the visual system has no direct access to any motion stimulus, only the responses of neural mechanisms on which to base decisions, this approach highlights the importance of considering mechanism-based explanations of global motion phenomena.

The presentation of moving stimuli within multiple apertures provides a useful tool for studying the factors that determine how local motion estimates are integrated across space, in both artificial and naturalistic stimuli (e.g., Mingolla et al., 1992; Alais et al., 1998; Kane et al., 2009, 2011). For example to probe the nature of the pooling process underlying global motion perception, Amano et al. (2009) developed a novel multi-aperture hybrid stimulus that contains aspects of both narrowband stimuli (e.g., plaids) used in conventional studies of global motion and also the multi-element nature of RDKs. In a series of psychophysical experiments they showed that when a moving surface consists of arrays of either Gabors (1-d) or plaids (2-d) moving behind fixed, spatially restricted apertures, the visual system employs different computations for pooling 1-d and 2-d local motion signals. According to Amano et al. (2009), the spatial integration of 1-d motion information conforms to the IOC rule, whereas the spatial pooling of local 2-d motion signals follows the VA of the physical velocities present in the stimulus. The authors concluded that spatial motion integration exhibits great flexibility and the strategy employed by the human visual system to encode global motion is dependent on the stimulus characteristics. Most importantly, for 2-d stimuli in which there is no local directional ambiguity (i.e., plaid micropatterns for which the aperture problem can be solved at each location in space) perceived direction appeared to be broadly consistent with a VA strategy of those 2-d directions across space. This result appears to be in stark contrast to that found by Webb et al. (2007), which suggested that for spatially 2-d moving stimuli such a random dots a neural-based explanation, based on say a ML or WTA computation, might be the key to understanding the nature of the pooling process under a wide-range of conditions. Indeed Webb et al. (2011) have subsequently shown that caution should be exercised before concluding that changes in behavior on a task, driven by changes in the stimulus configuration, is evidence of a flexible motion pooling system that uses different computations under different circumstances. They found that a single, ML computation could accommodate the apparently flexible nature of spatiotemporal motion pooling using RDKs in humans.

Thus, considerable uncertainty still remains concerning the computations that govern perceived global direction in human vision. The present study aimed to reconcile the disparate results reported by Webb et al. (2007) and Amano et al. (2009), and clarify the nature of the spatial pooling of local 2-d motion signals, by conducting two experiments that incorporated key elements of both of these studies. In Experiment 1 we used a novel multi-element stimulus consisting of spatially 2-d (isotropic), broadband noise textures that moved behind fixed, spatial apertures. This allowed us to determine if differences in perceived global motion reported in previous studies depend critically on the choice of stimulus-based characteristics (i.e., constraining local motions to a set of sparse spatial apertures). To test whether or not the local ambiguity of the direction information contained within each region of space is the principal factor that governs the computation underlying perceived global direction, in Experiment 2 we systematically varied the fidelity of the texture motion within each spatial aperture. In addition for each experiment we compared the psychophysical performance of the human observers to the performance of a neural population-decoding model utilizing either a VA, ML, or WTA algorithm.

## Materials and methods

### Observers

Five observers took part in the experiments. Observer FR was one of the authors and the remaining observers (AA, LX, KH, and ZH) were all naïve to the purpose of the study. All had normal or corrected-to-normal acuity and no history of ocular ill health. Informed consent was obtained from all subjects and experimental procedures met the ethical guidelines of the School of Psychology at the University of Nottingham.

### Apparatus and stimuli

Global motion stimuli were computer generated and displayed on a *LaCie Electron 22blue* CRT monitor with a spatial resolution of 1024 × 768 pixels and at a frame rate of 75 Hz. The monitor was carefully gamma-corrected with a spot photometer (*Konica Minolta LS-110*) and internal look up tables. Psychophysical procedures were also used to confirm that any residual luminance non-linearities were minimized (Ledgeway and Smith, 1994). The mean luminance of the display was 25 cd/m^2^. Viewing was binocular and from a distance of 76.3 cm, such that one screen pixel subtended 1.35 arc min of visual angle.

**Figure 1:**
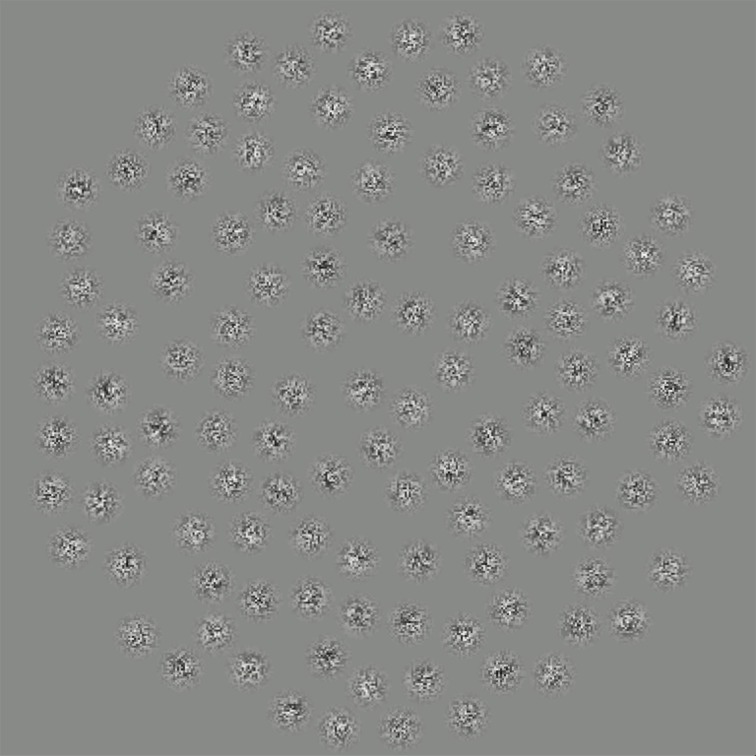
**Illustration of the multi-element stimulus used to investigate the computations that govern performance on tasks that require human subjects to pool local visual motion signals across space.** The stimulus consisted of spatially 2-d (isotropic), broadband noise textures that moved behind fixed, spatially distributed apertures. Observers judged which of two sequentially-presented stimuli (*standard* or *comparison*) moved in a more clockwise global direction of motion. The *standard* was composed of noise textures all moving rigidly in the same direction. For the *comparison* stimulus the individual texture directions on each displacement were sampled (with replacement) from probability distributions designed to distinguish between different global motion encoding strategies.

The motion stimuli used were similar in principle to those employed in previous studies of “motion-defined” contours (e.g., Ledgeway and Hess, 2002) and were presented within the confines of a circular display window (diameter 17.28°). Each stimulus consisted of a random spatial array of multiple patches of isotropic, uniformly distributed (spatially 2-d) noise, presented on a uniform background of mean luminance (see Figure 1). Each patch was composed of binary random noise elements (0.05° square) presented within a smooth stationary Gaussian spatial envelope (*SD* 0.23°, truncated at ± 0.56°). The Michelson contrast of the noise texture within each aperture was 0.99. The noise texture within each aperture could be either displaced (Experiment 1), or replaced with another stochastic sample (Experiment 2), at an update rate of 37.5 Hz. On each update the noise could be made to move in any desired direction, independently of both its previous displacements and those of neighboring apertures in the image, at a drift speed of 4.64°/s. The minimum center-to-center spacing between apertures was 1.13° and all noise textures were presented for 530 ms.

### Procedure

A two-alternative forced-choice (2AFC) task was employed. On each trial observers were presented with a central fixation cross, followed by the sequential presentation of two motion stimuli (each for 530 ms), separated by an inter-stimulus interval of 500 ms. One of the stimuli contained noise textures that all moved in the same direction (termed the *standard*) and the other (termed the *comparison*) contained noise textures whose directions were sampled from an underlying probability distribution (see Direction distributions below). The order of presentation of the *standard* and the *comparison* stimuli was randomized on each trial. The observers were required to judge the angular difference between the global directions of the two global noise texture stimuli (*standard* and *comparison*) and identify the one that moved in the more clockwise (CW) global motion direction. For example if the stimulus presented first on a given trial was perceived as translating (say) vertically upwards, equivalent to 12 o'clock, and the second stimulus toward 1 o'clock, then the observer would respond that the stimulus in the second interval had the most CW direction. For both experiments the global direction of the *comparison* stimulus on each trial could be either the same, slightly more CW or slightly more counter-clockwise (CCW) than the *standard*. This was done using the method of constant stimuli, in which the magnitude of the angular direction difference between the two stimuli on each trial was randomly chosen from a set of nine predetermined values (selected on the basis of pilot studies to bracket the Point of Subjective Equality—PSE). This procedure effectively avoids systematic order effects whilst allowing the experimenter full control of the stimulus set presented. Following the observer's response the fixation cross was presented for 500 ms before the next trial commenced.

Each observer completed a minimum of four runs of 90 trials for each condition tested and the resulting data were used to derive a psychometric function, expressing the percentage of trials on which the *comparison* was judged to be more CW than the *standard* as a function of the angular difference between them, as illustrated in Figure 3. Each psychometric function was fitted with the following logistic function in order to estimate the PSE (the angular difference at which the observer judged the two motion stimuli to have the same perceived direction):

(1) $$P_{cw}=100/(1+\text{exp}[(x-a)/b]),$$

where *P*_*cw*_ is the percentage of *comparison* CW responses, *x* is the direction difference between the global noise texture stimuli, *a* is the PSE and *b* is an estimate of the slope of the function at the inflection point.

### Direction distributions

**Figure 2:**
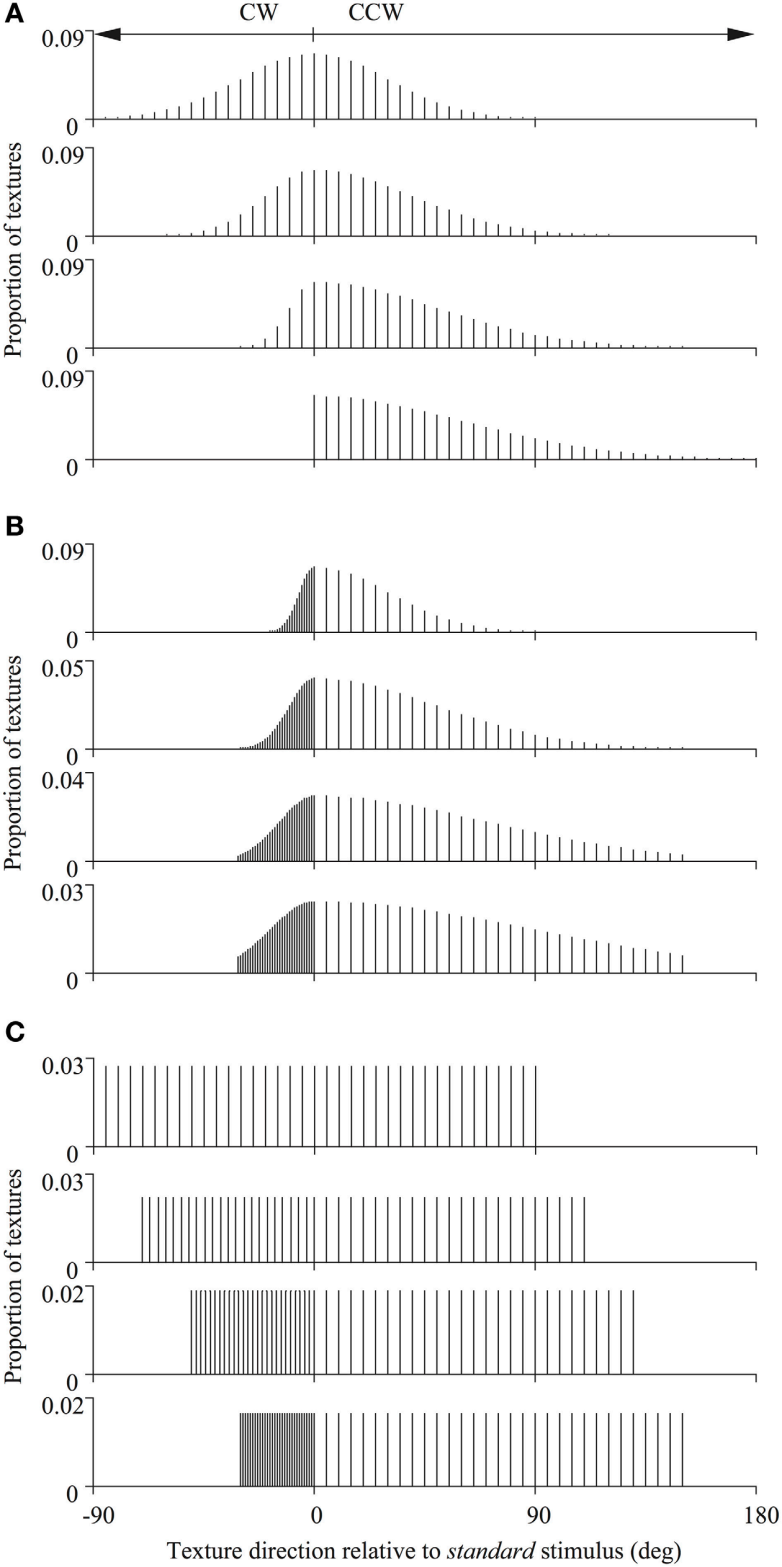
**Distributions of texture directions used for the different *comparison* stimuli.** The directions of the global noise textures were drawn from asymmetric Gaussian **(A,B)** or uniform **(C)** probability distributions. The half-widths and sampling densities of the counter-clockwise (CCW) and clockwise (CW) halves of the distributions (relative to the modal direction for Gaussian distributions and the median direction for uniform distributions) were varied to distinguish between different global motion encoding schemes. These probability distributions have been used previously in studies of global motion employing RDKs and are fully documented elsewhere (see Webb et al., 2007, 2011).

**Figure 3:**
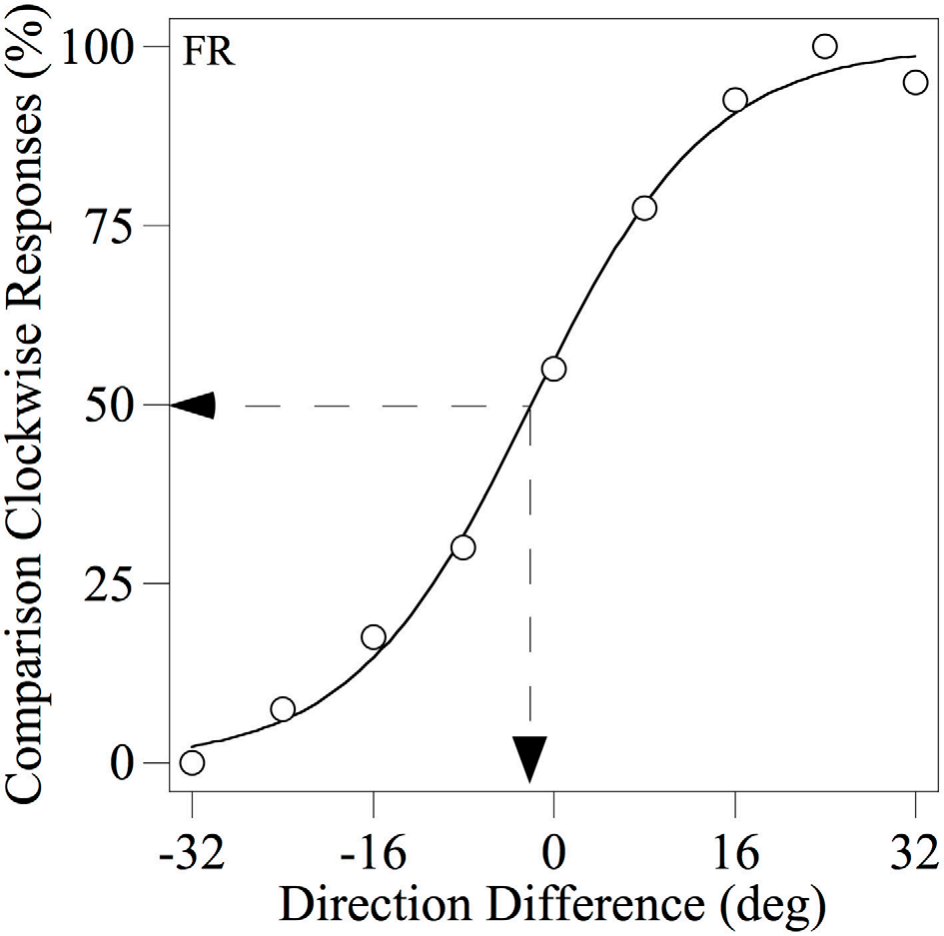
**Example psychometric function for observer FR showing the percentage of trials on which the *comparison* was judged as more clockwise than the *standard* as a function of the difference between their modal directions.** The smooth line through the data points is the best-fitting logistic function based on Equation 1. The dotted lines indicate how the PSE (angular difference at which the two motion stimuli were judged to have the same perceived direction) was derived.

In Experiment 1 the *standard* stimulus was composed of all local noise textures moving rigidly in the same direction (randomly chosen, on each trial, from the 360° range). Whereas, on each displacement, the individual texture directions of the *comparison* stimulus were sampled discretely, with replacement, from one of a set of 12 skewed probability distributions (with either a Gaussian or a rectangular profile) with a total range of 180 deg (see Figure 2). Although the texture within each spatial aperture could therefore change its motion direction frequently during a single trial, the stimulus nonetheless appeared to drift *en masse* in a global direction as it does under analogous circumstances using RDKs (Williams and Sekuler, 1984). The global direction of each of these 12 probability distributions could be set to any desired value so that it was either the same or different to that of the *standard* stimulus on each trial, in order to generate psychometric functions like that illustrated in Figure 3. These probability distributions, collectively, were designed to distinguish between different global motion encoding schemes (i.e., stimulus-based statistics vs. neural-based population decoders). We independently varied the half-widths and sampling densities of the CCW and CW halves of the distributions (relative to the modal direction for Gaussian distributions and the median direction for uniform distributions). We have used these particular distributions previously in analogous studies using RDKs and their construction and characteristic properties are extensively documented elsewhere (for details see Webb et al., 2007, 2011).

In Experiment 2 the fidelity of the texture motion within each spatial aperture was manipulated to investigate if the local ambiguity of the direction information contained within each region of space could govern the computation underlying perceived global direction. On each displacement, a percentage of the apertures in the *standard* stimulus (either 25, 37.5, 75, or 100%) contained textures that moved rigidly in a common direction whereas the remainder contained dynamic visual noise (that was uncorrelated over time) to create locally ambiguous motion. Similarly in the *comparison* stimulus a percentage of the apertures (same as the *standard*) contained textures with directions sampled (with replacement), on each displacement, from a probability distribution that was diagnostic for distinguishing between VA and ML (or WTA) predictions. The remaining apertures contained dynamic uncorrelated noise that created locally ambiguous motion. The proportion of apertures manipulated in this manner, which we term the aperture coherence, was kept the same, across trials, between *standard* and *comparison* stimuli.

### Model predictions

**Figure 4:**
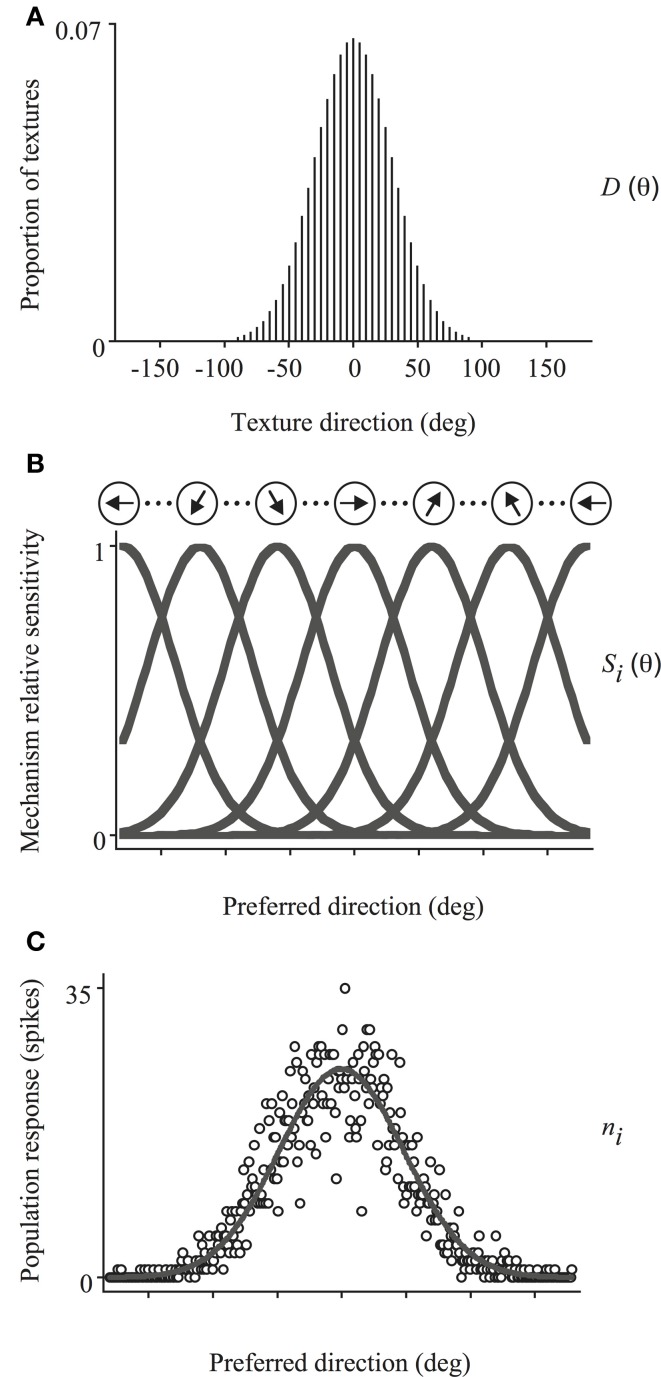
**Schematic illustration of the model used to simulate trial-by-trial performance on the global direction discrimination task.** The set of directions present in the stimulus **(A)** activates a bank of direction-tuned mechanisms, each with a Gaussian sensitivity profile corrupted by Poisson noise **(B)**. The population response **(C)**, is then decoded using either a VA, ML, or WTA read-out algorithm to identify the global direction of motion. See text for further details.

We simulated the integration of local directions by reading out the responses of a set of motion-sensitive mechanisms, with biologically-plausible properties akin to neurons found in visual cortex (e.g., MT), using VA^2^, ML and WTA decoders. This model has been described in detail elsewhere (e.g., Webb et al., 2007) and is depicted schematically in Figure 4. Briefly, it is composed of a set of evenly spaced direction-tuned mechanisms spanning the 360° range, each with a Gaussian sensitivity profile to motion direction (half-width, half-height bandwidth of 45°) and response corrupted by Poisson noise. The sensitivity of the *i*th mechanism, centered on directionn θ_*i*_, is given by:

(2) $$S_{i}(\theta )=\exp\{-{\left[(\theta -\theta_{i})/h\right]}^{2}\log2\},$$

where *h* is the tuning bandwidth (Figure 4B). The response of the *i*th mechanism to a moving texture stimulus *D* (see Figure 4A) with a distribution of directions, *D*(θ), is then:

(3) $$R_{i}(D)=R_{\max}t\sum_{\theta =\;1}^{360}S_{i}(\theta )pr\{D(\theta )\},$$

where *R*_max_ is the maximum mean firing rate of the mechanism (fixed at 60 spikes/s), *t* is the duration of the stimulus and *pr*{*D*(θ)} is proportion of texture directions in the probability distribution. The number of spikes (*n*_*i*_) evoked by a stimulus is Poisson distributed with a mean of *R*_*i*_(*D*), similar to the firing statistics of many cortical neurons which typically exhibit Poisson-like variability (e.g., Softky and Koch, 1993; Shadlen and Newsome, 1998).

(4) $$p(n_{i}|D)=\frac{R_{i}{\left(D\right)}^{n}i}{n_{i}!}\exp\{-R_{i}(D)\},$$

The pattern of neuronal activity across the population of mechanisms in response to a given stimulus, as exemplified by Figure 4C, is then decoded using either a VA, ML, or WTA read-out algorithm to identify the global direction of image motion. VA estimates perceived global direction by averaging the preferred directions of all mechanisms weighted by the magnitude of their respective responses:

(5) $$VA=\tan^{-1}\left(\frac{\sum_{i=1}^{360}n_{i}\sin\;(\theta_{i})}{\sum_{i=1}^{360}n_{i}\cos\;(\theta_{i})}\right),$$

ML estimates perceived direction from the weighted sum of responses of the population of mechanisms by multiplying the activity of each mechanism by the log of its tuning function (e.g., Jazayeri and Movshon, 2006):

(6) $$\log L(D)=\sum_{i=1}^{360}n_{i}\log R_{i}(D),$$

ML direction is then given by the value of θ_*i*_ for which log *L*(*D*), computed for all *D*, is maximal. Although the ML calculation is identical to that used in our previous work, in which it disambiguates neural activity related to Poisson variability, it is certainly not optimal under circumstances when sources of ambiguity also arise in the stimulus (e.g., from the presence of stimulus-based noise or from the existence of multiple directions in the scene). Although that ML computation was initially tailored to deal with coherent motion throughout the visual scene, we utilize it in the present paper because it allows us to investigate the general structure of the neural computation underlying global motion perception and compare its performance with that of the VA and WTA algorithms, across a range of conditions, using the same model architecture.

WTA calculates perceived global motion as the preferred direction of the most active mechanism. That is, the value of θ_*i*_ for which *n*_*i*_ in the population response is maximal.

We simulated each model's performance on the direction discrimination task, on a trial-by-trial basis, using analogous methods to those used in the psychophysical experiments. It is important to note that space is not represented explicitly in these simulations and the inputs to the model were not actual instantiations of visual images, but simply the sets of directions that would normally be assigned to the textures of the *standard* and *comparison* stimuli on each trial (those for the latter were generated by sampling, with replacement, the probability distributions depicted in Figure 2). In the case of Experiment 2, whenever an aperture contained dynamic uncorrelated noise (ambiguous motion), this was assumed to contribute equal motion energy in all directions and consequently activate all motion-sensitive mechanisms in the model uniformly to a small degree.

## Results

### Experiment 1

**Figure 5:**
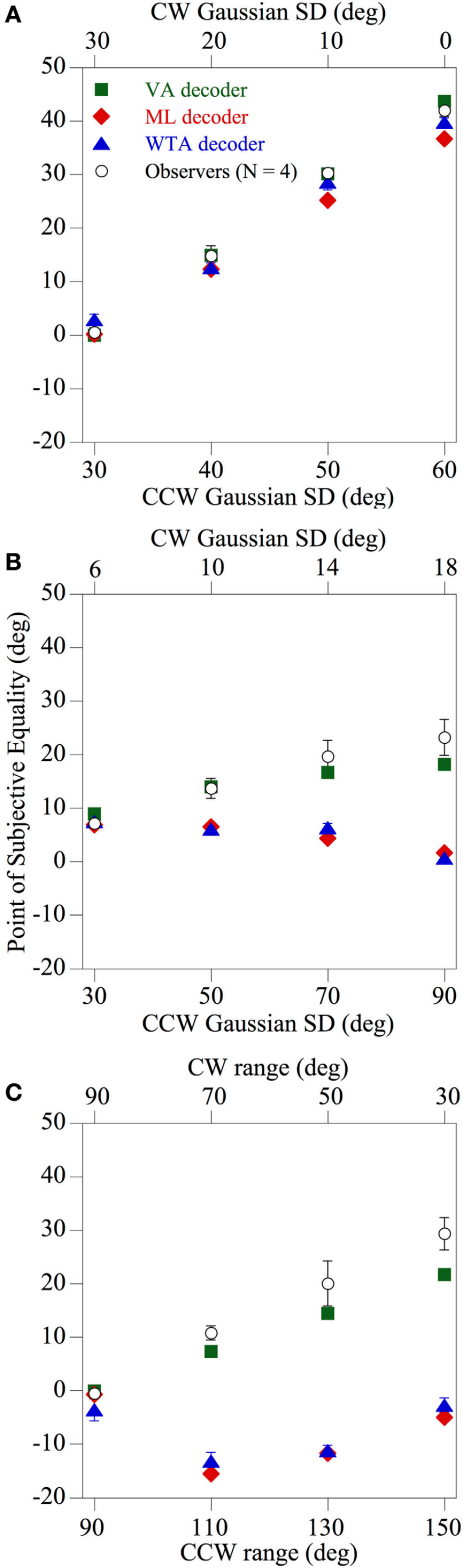
**Results of Experiment 1 showing the perceived global direction (averaged across four observers) and the corresponding model estimates derived from the three population decoders, indicated by different symbols, as a function of skew for each of the 12 *comparison* distributions tested.** The directions of the global noise textures were drawn from asymmetric Gaussian **(A,B)** or uniform **(C)** probability distributions, and the overall pattern of performance of the observers is most compatible with a VA decoder. The error bars above and below each data point represent ±1 SE.

Figure 5 shows how the mean perceived global direction of the observers (*N* = 4) and the corresponding estimates derived from the three population decoders, indicated by different symbols, changed as a function of skew for each of the 12 *comparison* distributions tested. It is readily apparent that the pattern of psychophysical results found using spatially-windowed arrays of moving noise textures differs markedly from that found using conventional RDKs (Webb et al., 2007). Indeed irrespective of whether the directions of the global noise textures were drawn from asymmetric Gaussian (Figures 5A,B) or uniform (Figure 5C) probability distributions, overall the performance of the observers was most consistent with that of the VA decoder [mean square error (MSE) between the perceived and model predictions was 12.83]. This was especially evident for the case of the *comparison* distributions used in Figures 5B,C, where the predictions of the ML and WTA population decoding algorithms diverged by as much as 34° away from the results obtained with the human observers (MSE 335.53 and 304.91, respectively) compared with only 7.6° for the VA prediction. Thus, the results are broadly consistent with those of Amano et al. (2009), in that the integration of 2-d local motion signals across space appears to be governed by a VA computation, when those motions are confined to set of fixed spatial apertures.

### Experiment 2

**Figure 6:**
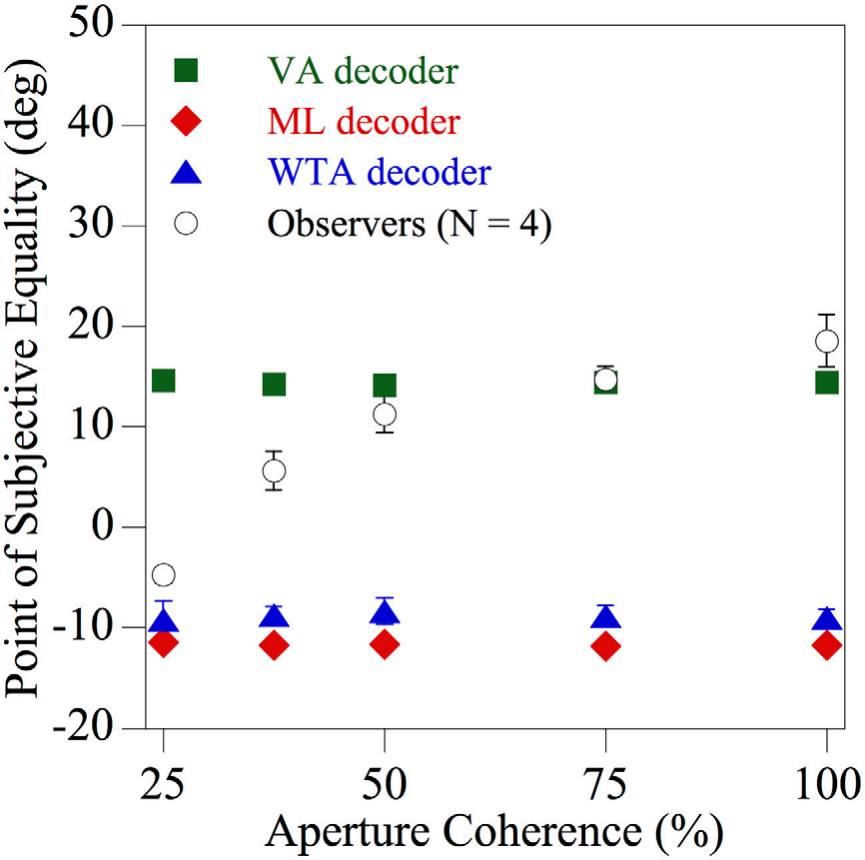
**Results of Experiment 2 showing the perceived global direction (averaged across four observers) and the corresponding model estimates derived from the three population decoders, indicated by different symbols, as a function of the percentage of apertures in the stimulus that contained coherent texture directions.** For the *comparison* stimulus the individual texture directions on each displacement were sampled (with replacement) from one of the probability distributions used previously in Figure 5C (CCW range of 130° and CW range of 50°) that was diagnostic for distinguishing between VA, ML, and WTA estimates. The error bars above and below each data point represent ±1 SE.

To investigate whether or not the local ambiguity of the direction information contained within each region of space influences the computation underlying perceived global direction, the fidelity of the texture motion within each spatial aperture was manipulated. Dynamic, but uncorrelated, visual noise was presented within a proportion of the apertures to create locally ambiguous motion. The results for one of the diagnostic skewed (uniform) *comparison* distributions used previously in Figure 5C (with a CCW range of 130° and a CW range of 50°) are shown in Figure 6. The mean perceived global direction of the observers (*N* = 4) and the corresponding estimates derived from the three population decoders, indicated by different symbols, are plotted as a function of the aperture coherence (percentage of apertures in the stimuli that contained coherent texture directions). When the motion signals within the spatial apertures consisted of only coherent texture directions (100% aperture coherence), observers' global motion perception once again closely followed the VA prediction. However, when more than half of the apertures contained uncorrelated dynamic noise (i.e., aperture coherence <50%), creating substantial local ambiguity, the perceived global direction shifted progressively toward that of the ML and WTA population decoder predictions, which were similar. Hence the results of this experiment clearly show that global motion perception is dramatically affected by the local ambiguity of the direction information contained within each aperture.

## Discussion

The experiments conducted in the current study were designed to further explore the nature of the principles governing the spatial pooling of 2-d motion signals in human vision. In particular we were motivated to reconcile recent contrasting findings that have led to disparate ideas on how the visual system might encode the global direction of image motion. As discussed previously, Amano et al. (2009) in a comprehensive examination of this issue presented evidence that the integration of local 2-d motions across space, conforms closely to the VA rule applied to the stimulus motions. However, Webb et al. (2007, 2011) reached a different conclusion. They demonstrated using RDKs that the spatial pooling of motion information is in general not well characterized by any stimulus-based metric, including the VA rule. Instead they showed that a neural mechanism-based solution, such as a ML or WTA computation, was a much better predictor of the psychophysical performance. A potentially critical difference between these sets of studies lies in the choice of stimuli, display configuration and methods. Amano and colleagues used novel arrays of narrowband plaids moving behind stationary, Gaussian apertures and only considered stimulus-based solutions to global motion such as the IOC and the VA of the physical motions present in the image. Furthermore, when investigating the spatial pooling of 2-d local motions, Amano et al. based their conclusions on measures of perceived global speed and did not measure perceived global direction directly. In contrast we (e.g., Webb et al., 2007) used conventional broadband RDKs, measured perceived global direction explicitly and compared psychophysical performance to mechanism-based predictions, based on decoding the responses of a biologically-inspired model of motion processing. Hence in the present study we sought to incorporate elements of both studies in an attempt to reconcile these very different approaches and reveal the nature of the pooling process underlying global motion perception.

In Experiment 1, we investigated if presenting rigidly-moving, spatially 2-d broadband noise textures within fixed, spatial apertures was sufficient to produce a different pooling strategy from that suggested by Webb et al. (2007) using RDKs. The results (Figure 5) showed that this was indeed the case and psychophysical performance under these conditions closely mirrored those of Amano et al. (2009), in that the perceived global motion direction coincided with the VA of the local motions within the stimulus (a VA read-out algorithm applied to a model neural population of motion-sensitive mechanisms produces the same prediction). However, this manipulation in itself does not explain why such gross differences in stimulus configuration, should lead to a qualitative change in the nature of global motion processing.

To address this issue further we designed an additional experiment to manipulate the fidelity of the motion present within each spatial aperture. In Experiment 2 we sought to explore whether or not the local directional ambiguity that characterizes each region of space within the stimulus is a key factor in determining the computational strategy used by the visual system to pool visual motion signals. That is, the human visual system could exhibit some degree of flexibility with regard to motion pooling and be able to exploit different solutions under different circumstances, as suggested by Amano et al. (2009) for spatially 1-d and 2-d patterns. It is important to note that even when spatial apertures are not present within a display, any spatially local region of a stochastically moving image such as the random-walk RDKs used by Webb et al. (2007, 2011), will necessarily contain a range of dot directions and hence at least some degree of directional ambiguity. The results of this experiment clearly showed marked differences between spatial integration in the presence of ambiguous local direction signals and unambiguous visual motion pooling. That is, a VA decoder accurately predicted the perceived global direction of image motion when the vast majority of apertures contained rigid texture movement (Figure 6). However, as the percentage of ambiguous motion present in the stimulus was progressively increased (i.e., dynamic uncorrelated noise was introduced to more apertures on each image update), the observers' global direction judgments shifted toward the ML and WTA predictions. It is important to reiterate, however, that we are not claiming that the ML scheme implemented in the present paper is an optimal decoding strategy under the current circumstance, as the model was not tailored to the specific stimuli employed and does not take into account the considerable directional ambiguities arising from the stimuli, especially in Experiment 2. However, we were interested in the generality of the ML algorithm, that we and others have previously applied to RDKs (e.g., Jazayeri and Movshon, 2006), and how it compares to the behavior of other (non-optimal) algorithms such as the VA, that are typically used to explain global motion processing, when implemented within a common, biologically-inspired, framework.

It is tempting to explain these findings in terms of an adaptive process that switches between a VA and a ML (or alternatively a WTA) computation depending on the degree of local ambiguity or directional uncertainty present in the stimulus. That is, the human visual system could exhibit some degree of flexibility with regard to motion pooling and is able to exploit different solutions under different circumstances. Such a general notion is not incompatible with the response properties of some neurons in macaque MT (Pack and Born, 2001; Smith et al., 2005; Majaj et al., 2007), task-dependent modulations of following and pursuit eye movements (Recanzone and Wurtz, 1999; Ferrera, 2000; Barthelemy et al., 2010) and recent MEG and fMRI investigations of the responses of the human homologue of MT (hMT+) to spatially-distributed 1-d motion signals (Amano et al., 2012). This does, however, raise the issue of actually how the visual system selects the motion pooling strategy that is best suited to a particular stimulus and task. Furthermore, we have previously shown (Webb et al., 2011) that changes in performance on global motion tasks, resulting from changes in the stimulus configuration, are not necessarily indicative of a flexible pooling system that uses different computational strategies under different circumstances. Indeed it is possible that this behavior is consistent with a generalized population vector decoding algorithm that applies a power-law non-linearity to the neural response (spike count) of each mechanism prior to pooling those responses. For example Wei and Stocker (2012) have recently shown that such a framework defines an entire class of decoding mechanisms that includes both the VA read-out and the WTA decoder, depending on the magnitude of the exponent of the power-law non-linearity. We are currently exploring some of these issues in our laboratory.

### Conflict of interest statement

The authors declare that the research was conducted in the absence of any commercial or financial relationships that could be construed as a potential conflict of interest.

## Footnotes

^1^Note that for simplicity the term “VA” will be used to indicate both vector average and vector sum (the two algorithms only differ in terms of their predictions concerning the perceived speed of global image motion and not perceived global direction).

^2^Webb et al. (2007) showed that predictions of perceived global direction estimated from a model-based VA decoder are very similar to those obtained by simply computing the vector average of the local physical directions within the stimulus (cf. Amano et al., 2009).
